# Air Pollution and microRNAs: The Role of Association in Airway Inflammation

**DOI:** 10.3390/life13061375

**Published:** 2023-06-12

**Authors:** Fabiana Furci, Alessandro Allegra, Alessandro Tonacci, Stefania Isola, Gianenrico Senna, Giovanni Pioggia, Sebastiano Gangemi

**Affiliations:** 1 Allergy Unit and Asthma Center, Verona University Hospital, 37134 Verona, Italy; gianenrico.senna@univr.it; 2 Division of Hematology, Department of Human Pathology in Adulthood and Childhood “Gaetano Barresi”, University of Messina, 98124 Messina, Italy; alessandro.allegra@unime.it; 3 Clinical Physiology Institute, National Research Council of Italy (IFC-CNR), 56124 Pisa, Italy; alessandro.tonacci@cnr.it; 4 School and Operative Unit of Allergy and Clinical Immunology, Department of Clinical and Experimental Medicine, University of Messina, 98124 Messina, Italy; stefaniaisola.si@gmail.com (S.I.); gangemis@unime.it (S.G.); 5 Department of Medicine, Verona University Hospital, 37134 Verona, Italy; 6 Institute for Biomedical Research and Innovation (IRIB), National Research Council of Italy (CNR), 98164 Messina, Italy; giovanni.pioggia@irib.cnr.it

**Keywords:** microRNA, epigenetics, lung, inflammation, air pollutants, smoke, airway diseases

## Abstract

Air pollution exposure plays a key role in the alteration of gene expression profiles, which can be regulated by microRNAs, inducing the development of various diseases. Moreover, there is also evidence of sensitivity of miRNAs to environmental factors, including tobacco smoke. Various diseases are related to specific microRNA signatures, suggesting their potential role in pathophysiological processes; considering their association with environmental pollutants, they could become novel biomarkers of exposure. Therefore, the aim of the present work is to analyse data reported in the literature on the role of environmental stressors on microRNA alterations and, in particular, to identify specific alterations that might be related to the development of airway diseases so as to propose future preventive, diagnostic, and therapeutic strategies.

## 1. Introduction

Many airway diseases can be considered to be the result of gene activity, environmental factors, and the interactions between them. However, as protein-coding genes cannot always explain all reported genomic effects, in recent years, the study of epigenetic effects and non-coding gene products has advanced in terms of the evaluation of the pathogenesis of many diseases. MicroRNAs (miRNAs) are small, noncoding RNAs that play an essential role in post-transcriptional gene expression. They are involved in many pathophysiological processes, such as stem cell differentiation, haematopoiesis, cardiac and skeletal muscle development, neurogenesis, insulin secretion, cholesterol metabolism, and the immune response, as well as in the pathogenesis of cancer, asthma, and other diseases [1,2].

The correlation of such diseases with distinct miRNA profiles highlights the key role of specific miRNA programs in the activation of pathophysiological processes, not only in terms of diagnosis but also in terms of future studies and therapeutic approaches [3]. Nowadays, we hear more and more about the exposome, which, unlike the genome, is highly variable and includes all environmental exposures and factors from the prenatal period onward. Climate and urban dwelling determine exposome composition, which is made up of pollutants, microbes, and allergens that, above all, affect the homeostasis of the respiratory mucosae [4].

Among chronic respiratory diseases, air pollution and climate change significantly play a key role in Th2 inflammation, as occurs in allergic rhinitis (AR) and asthma. Outdoor air pollution is produced by traffic and other human activities, acting negatively both directly on human health and by enhancing the allergenicity of some plants. In particular, climate change plays a key role in inducing modifications in the availability and distribution of plant- and fungal-derived allergens, as well as increasing the occurrence of extreme climate events [5]. However, the relationships among chronic respiratory diseases can be better understood if we consider that such conditions share many pathophysiological factors whose prevalence is constantly increasing in Westernized lifestyles worldwide [5,6,7].

## 2. miRNA: General Consideration

The biogenesis of microRNA (miRNA), small non-coding miRNA molecules, begins in the nucleus by transcription of the primary miRNA (pri-miRNA), which is an approximately 500–3000-base-long molecule produced by transcription of the miRNA gene or intron by RNA polymerase II or III. Pri-miRNA is cleaved to an approximately 70-base-long precursor miRNA (pre-miRNA) by the Drosha–Pasha (DGCR8) complex. Subsequently, in cytoplasm, the export of pre-miRNA from the nucleus by the protein exportin 5 occurs. The cleavage of a pre-miRNA hairpin to the miRNA duplex form is mediated by the RNase III enzyme Dicer, with the production of a double-stranded RNA duplex that is modified into a functional, mature single-stranded form of miRNA together with Argonaute proteins. Production of the RNA-induced silencing complex (RISC) with mature miRNA follows [8,9]. miRNAs, regulators of development in worms and fruit flies, have a key role in the pathogenesis of human diseases. Regarding the respiratory system, miRNAs play a key role in normal pulmonary development and in maintaining lung homeostasis [10].

miRNA functions can be regulated by a number of mechanisms occurring, for example, during transcription, miRNA processing, and target interaction [11]. At the transcription level, various regulatory factors that are involved in the direct binding to miRNA promoter elements and control of their expression have been identified. In this regard, there is evidence that the tumour-suppressor gene p53 binds to the promoter regions of the miR-34 family, which are involved in inducing in vitro cell cycle arrest and apoptosis [12,13,14]. miRNA transcription, dependent on methylation status, is also influenced by many epigenetic factors. Moreover, there is extensive methylation of the let-7a locus in normal lungs; on the other hand, in some patients affected by lung adenocarcinoma, there is hypomethylation of the let-7a locus. Therefore, epigenetic factors may have a relevant role in the development of malignant diseases [15].

In the literature, it has been reported that specific miRNA expression profiles play a key role in lung development and homeostasis [16,17]. Many foreign particles act on the respiratory epithelium, such as air pollution, toxins, and potential pathogens, against which human immunological mechanisms intervene. In this framework, miRNAs are essential both in adaptive and innate immunological mechanisms [18,19].

## 3. Tobacco Smoke and miRNAs in Airway Diseases

**Figure 1:**
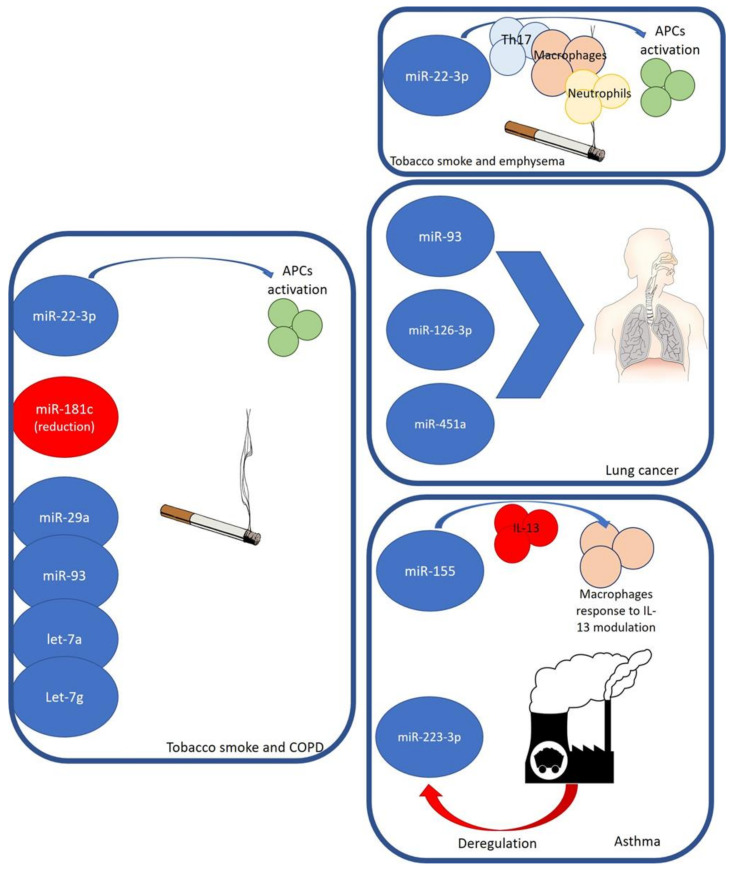
miRNAs, environmental factors, and relationships with some airway diseases.

Tobacco smoke, containing numerous toxic chemical compounds, such as oxidative gases, particulate matter (PM), carcino-genes, and heavy metals, induces many effects on the airway health of non-smokers who inhale smoke, even in the form of second-hand smoke (SHS) exposure [20] (Figure 1).

SHS prenatal and postnatal exposure was related to an increased risk of asthma during childhood, in particular, in children that are exposed to tobacco smoke during the first two years of life [21,22]. Moreover, SHS exposure may be related to epigenetic changes with consequent repercussions on the development of asthma [23]. The greater susceptibility of children to SHS could be due to the immaturity of their immune and respiratory systems and the larger air volume per kilogram of weight inhaled compared to adults [24]. In the respiratory airways, there is the colonisation of bacteria, viruses, and fungi, which can be modified after exposure to many environmental factors (e.g., tobacco smoke), with the appearance of a state of bacterial dysbiosis, in particular, with the overproduction of *Staphylococcus aureus*, upregulating airway mucus production, impairing mucociliary clearance, and inducing lung inflammation that promotes airway remodelling [25,26,27]. In particular, in the literature, it has been reported that smoke exposure induces a down-regulation in miRNAs, which was observed for 23 miRNAs in humans, 24 miRNAs in rats, and 15 miRNAs in mice. On the other hand, smoke-related up-regulation was seen for only 5 miRNAs in humans, 1 miRNA in rats, and none in mice [28,29,30]. Furthermore, it has been demonstrated that 13 miRNAs that were down-regulated in mouse lungs were also down-regulated in rat lungs; miR-30b and miR-43 were down-regulated in mice only, and let-7c, miR-10a, miR-30a, miR-34c, miR-123, miR-145, miR-146, miR-191, miR-219, miR-222, and miR-223 were down-regulated in rats only [28,29]. miR-30, miR-99, and miR-125, which were down-regulated in both mice and rats, and miR-146 and miR-223 which were down-regulated in rats only, were also found to be downregulated in humans. Overall, the most strongly down-regulated miRNA in human smokers was miR-218 [29].

As reported by Izzotti et al., the regulation of miRNAs plays an essential role in the postnatal development and maturation of the lung. The reaction of pulmonary miRNAs to environmental agents and, in particular, to cigarette smoke (CS), the most significant human carcinogen, induces the activation of adaptive mechanisms and many pathways involved in the pathogenesis of pulmonary diseases, in which epigenetic and genotoxic mechanisms are involved. Moreover, specific variables, such as gender and age, would appear to influence the intensity of miRNA dysregulation in response to environmental factors. These results were obtained following analyses carried out by the same authors on miRNA expression patterns in the lungs of mice exposed to passive CS, comparing life-course-related miRNA expression changes in lungs of unexposed newborn, postweaning, and adult mice [30].

It is reported that quitting smoking induces an alteration of plasma miRNA profiles to resemble those of non-smokers, highlighting that the plasma miRNA differences between smokers and non-smokers may be induced or not by repeated cigarette smoking, although acute exposure of ex-smokers to CS did not induce a relevant alteration in the plasma miRNA profile [31]. Another important aspect is that smoking during pregnancy impacts health during childhood and later in life, indicating that epigenetic mechanisms are responsible for tissue-specific gene expression during differentiation, such as changes in chromatin structure through histone acetylation and methylation, coordinated changes in the methylation of cytidine–guanosine (CpG) nucleotides in the promoter regions of specific genes, and post-transcriptional control by microRNA [32]. Therefore, considering the sensitivity of miRNAs to environmental stressors, such as tobacco smoke, and their role in immunological pathways, such as regulatory T (Treg) cell differentiation, it has been reported that exposure to tobacco smoke during pregnancy is related to the level of miRNA-223 expression in maternal blood, with a consequent effect on children’s cord blood Treg cell numbers and the possible risk of allergic disorders. It has been highlighted that both maternal and cord blood miR-223 expressions were positively correlated with maternal urine cotinine levels, there was an association between maternal miR-223 expression and indoor concentrations of benzene and toluene, and an elevated expression of miR-223 was associated with lower levels of Treg cells in maternal and cord blood. The presence of lower Treg cell numbers at birth was related to an increased risk of atopic dermatitis during the first 3 years of life. Indeed, concentration of the toluene metabolite S-benzylmercapturic acid in maternal urine was associated with a decreased expression of miR-155 in cord blood [33]. The analysis of the possible role of air pollution exposure in prenatal life on placental miRNA expression reported that in utero PM2.5 exposure affects miRNAs expression, with an inverse association between placental expression of miR-21, miR-146a, and miR222 with PM2.5 exposure during the 2nd trimester of pregnancy, while there is a positive association between placental expression of miR-20a and miR-21 with 1st trimester exposure. Likewise, in the same analysis, the authors highlighted that tumour-suppressor phosphatase and tensin homolog (PTEN), which is a target of miRNAs, has a relevant association with PM exposure [34].

## 4. miRNA, Air Pollutants, and Airways Diseases

Dysregulation of miRNAs also plays a key role in chronic obstructive pulmonary disease (COPD). In the literature, elevated serum levels of miR-22-3p have been reported in smoker COPD patients compared with COPD patients exposed to biomass smoke. This miRNA-22-3p was related to the activation of antigen-presenting cells in the lungs in relation to tobacco smoke exposure [35]. It was seen that a reduction in miR-181c levels in lung tissues from COPD smoker patients emerged when compared with non-smoker COPD patients. From the analysis of 775 healthy subjects, cigarette smoking was associated with the expression of miR-29a, miR-93, let-7a, and let-7g using a machine-learning approach, highlighting that these molecules can be considered as potential serum biomarkers of environmental tobacco smoke exposure [36,37].

In vitro studies have reported the key role of the miR-22-HDAC4-IL-17 axis in the pathogenesis of emphysema in rats that were exposed to tobacco smoke through miR-22-activated lung-antigen-presenting cells (APC) after exposure to CS [38]. In particular, an upregulation of miR-22 in the lung APCs of smokers with emphysema was reported, which is not seen in patients without emphysema. In the lungs of CS-exposed rats with emphysema, increased expression of TH17 cells, macrophages, and neutrophils was seen. The gene that encodes histone deacetylase HDAC4 is a primary target of miR-22 in APCs [39].

An essential role is mediated by miR-93 in lung cancer, whose target genes are associated with transcription by MAPK1, RBBP7, and Smad7 [40]. Expression of miR-93-5p is reported in human subjects exposed to PM2.5 and CS. An association has been reported between miR-126-3p and asbestos and malignant mesothelioma [41,42]. The expression of miR-126-3p and miR-451 has been related to the severity of lung adenocarcinoma; in particular, showing low levels of miR-126-3p in the presence of large-diameter tumours and lymph node metastasis in lung adenocarcinoma. In human exposure studies, PM10 and ozone exposure were associated with the expression of the miR-126-3p molecule [43].

miR-155 plays a key role in various immunological and inflammatory processes [44]. It is crucially involved in the regulation of lung diseases, including asthma, tuberculosis (TB), sarcoidosis, and cystic fibrosis (CF). A reduced expression of miR-155 in the sputum of asthmatic patients has been reported compared to that in non-asthmatic patients. Moreover, there was a different expression of miR-155 during the allergy season, with reduced levels in asthmatic patients compared with allergic patients after the pollen season [45]. Mice with the miR-155 gene KO were reported to have lung remodeling and elevated leukocyte numbers in bronchoalveolar lavage fluid (BALF) after sensitization, with decreased IL-2 levels and IFN-g expression in comparison to sensitized mice without miR-155 KO, highlighting a protective anti-inflammatory function of miR-155 [46]. While a lack of miR-155 expression induces IL-4-stimulated Th2 cell differentiation, conversely, it has a preventive role in the activation of dendritic cell-triggered Th2 pathways in vivo [19,47]. miR-155 also plays a role in IL-13 that is related to inducing mucus production in asthma. In particular, in human macrophages, miR-155 acting on IL13R-alpha1 reduces levels of the IL13R-alpha1 protein, leading to diminished activation of signal transducer and activator of transcription 6 (STAT6). Therefore, miR-155 acts on the IL-13-dependent regulation of several genes (SOCS1, DC-SIGN, CCL18, CD23, and SERPINE) involved in the establishment of an M2/pro-Th2 phenotype [48].

miR-223-3p, a molecule involved in inflammatory processes, is overexpressed in neutrophils of patients with asthma. In particular, microRNA networks and genes are related to asthma severity, such as hsa-miR-223-3p, a neutrophil-derived microRNA, which plays a key role in TLR/Th17 signalling and endoplasmic reticulum stress [49]. Taking these data into account, deregulation of miR-223-3p following PM2.5 and ozone highlights the role of air pollutants in the development of many diseases other than cancer [50].

miR-155 plays a key role in Th2 inflammation by modulating the response of human macrophages to IL-13, implicated in allergic airway inflammation [51]. It is reported that the inhibition of miR-155 induces an increase in transcription factors involved in the generation of Th2 asthma inflammation. IL-13, downregulating the levels of miR-133a, and inducing increased expression of RhoA protein might be responsible for an increased contraction of bronchial smooth muscle [52,53,54,55].

In a mouse model of asthma, an increased expression of miR-21 and repression of miR-1 in IL-13 transgenic mice have been reported, in which there is an over-expression of IL-13 in a lung-specific manner. Increased levels of miR-21 may induce a reduction of IL-12, a macrophage-derived cytokine that is related to a pro-inflammatory phenotype [56].

In asthma, a downregulation of miR-1 has been reported that could induce smooth muscle hypertrophy and remodeling [57,58].

There have been reports of increased levels of miR-16, -21 and -126 in a mouse model of house dust mite-induced allergic asthma compared to control animals. The suppression of airway hyper-responsiveness to methacholine, reduction of the hypersecretion of mucus, and inhibition of the recruitment of eosinophils are described after the intranasal administration of antagomirs that inhibit miR-126 in a mouse model of allergic inflammation [59]. In the presence of increased levels of miR-146, airway smooth muscle cells and alveolar epithelial cells release IL-6 and IL-8 in a reduced way [60]. 

A study evaluating the expression of 227 miRNAs in bronchial biopsies of patients with mild asthma and non-asthmatic healthy controls reported no peculiar expression of miRNAs. Moreover, miRNA expression profiles are not influenced by corticosteroid treatment, and different airway cell types had different miRNA expression profiles [61,62].

In the literature, it is reported that miRNAs, through reactive oxygen species (ROS), are involved in epithelial-to-mesenchymal transition (EMT) induction. Endogenously generated ROS or those produced by environmental factors can modify miRNAs. Alternatively, miRNAs can directly participate in EMT, as occurs in the case of miR-21 in pulmonary epithelial cells. EMT is a complex process associated with cell transformation and plays a key role in many diseases, such as cancer. It can be inducted by crystalline silica Min-U-Sil 5 (MS5) with the consequent activation of a neoplastic-like cell transformation process, in which Glo1 is involved. Glo1 up-regulation induces AP-modified Hsp70 protein depletion, with consequent SMAD4 activation and miR-21-dependent SMAD7 inhibition [63].

**Figure 2:**
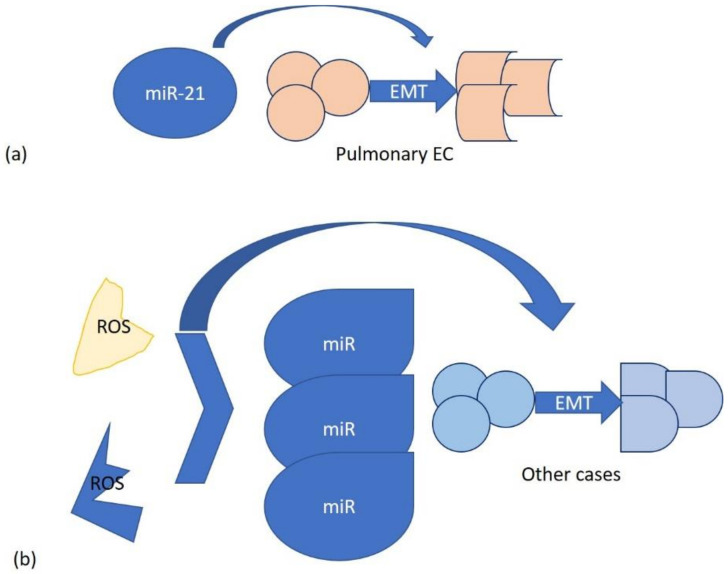
Two mechanisms of miRNA interaction in EMT: (**a**) specific, on pulmonary epithelial cells with miR-21; (**b**) generic, involving endogenous and environmental ROS.

Inhibition of Glo1 occurs at both functional and transcriptional levels, the latter through ERK1/2 MAPK and miRNA 101 involvement [64] (Figure 2).

## 5. The Use of miRNA as Therapeutic Strategy in Lung Disease

Studies about miRNAs have increased, above all regarding their possible use as novel therapeutic targets for the treatment of various diseases. The miR-17–92 cluster (which contains miR-17, -18, -19, -20, and -92) plays a key role in regulating lung development; indeed, expression is high in embryonic lung and is reduced through development into adulthood. In the field of respiratory medicine, it is reported that there is a relationship between the reduction in growth of both murine and human non-small cell lung tumours and the overexpression of let-7g from lentiviral vectors; reduced expression of let-7 family members is common in non-small cell lung cancer (NSCLC) [10,65]. Also, Trang et al. reported that exogenous delivery of let-7 to established tumours in mouse models of NSCLC induces a significant reduction of the tumour burden, highlighting the therapeutic potential of let-7 in NSCLC [66]. Considering some variables, such as the way of delivery, tissue specificity, and stability of the therapeutic strategies for the lung, therapeutic agents can be used via the intrapulmonary route or aerosolization [67]. RNA interference (RNAi) is a post-transcriptional gene-silencing method. Fujjita et al., in experimenting with a new class of RNAi therapeutic agents (PnkRNA™, nkRNA) characterised by high resistance to degradation, less immunogenicity, less cytotoxicity, and efficient intracellular delivery, developed a different platform to promote naked RNAi approaches administered through aerosol delivery in mice. Furthermore, the authors reported that a naked, unmodified novel RNAi agent, such as ribophorin II (RPN2-PnkRNA), used as a therapeutic target for lung cancer, plays a key role in the inhibition of tumour growth without any significant toxicity [68].

Considering that the degradation of free miRNAs is rapidly induced by nucleases in serum and extracellular fluids, anti-miRNA therapy is considered a potential therapeutic strategy for lung disease, as the oligonucleotides can be successfully delivered without the requirement of delivery vectors [69].

As many miRNAs have been implicated in the regulation of asthma inflammation, in one model, a toll-like receptor 4–induced Th2 lymphocyte induced high miR-126 expression, and a selective blockade of miR-126 plays a key role in the block of the asthmatic phenotype, inducing a reduction in TH2 responses, airway hyperresponsiveness, inflammation, mucus hypersecretion, and eosinophil recruitment [59]. However, more recently, extracellular vesicles (EVs), such as exosomes and microvesicles, which are released from many respiratory cells and contain various molecules, including proteins and miRNAs indicating the pathophysiological state of the origin cells, are taking on a growing role regarding both pathogenesis and therapeutic strategies in many lung diseases [70,71].

## 6. Future Perspectives

In the near future, more entities, such as circular RNAs and long, non-coding RNAs will need to be considered in research on how air pollution affects non-coding genetic material and respiratory disorders. [72]. Circular RNAs (circRNAs) are a different class of ncRNAs that perform essential pathophysiological functions relevant to the emergence of many diseases [73,74]. Back-splicing (head-to-tail splicing) is the process used to create circRNAs, which results in molecules with between 30 and 50 phosphodiester bonds [75]. CircRNAs carry out their biological functions using a variety of mechanisms, such as RNA polymerase II-dependent transcriptional initiation stimulation, parental exon-skipping induction due to competition with linear splicing, direct physical inhibition of protein function, miRNA sponging, and 5′Cap-independent initiation of protein translation [76].

Several studies have discovered a strong connection between respiratory illnesses, circRNAs, and air pollution. A study by Meng et al. showed that exosomal hsa circ 0005045 is upregulated by PM2.5 and binds to the protein cargo peroxiredoxin2, which functionally worsens the hallmarks of COPD by luring neutrophil elastase and inducing in situ release of tumour necrosis factor (TNF) by inflammatory cells. This was demonstrated using in vitro and in vivo PM2.5 exposure models. Using exosome transplantation and conditional circRNA-knockdown mouse models, the biological role of hsa circ 0005045 linked with COPD exacerbation has been confirmed. By classifying the main constituents of PM2.5, it was discovered that exosomal hsa circ 0005045 is elevated in response to PM2.5-bound heavy metals, which can be distinguished from the constituents of CS. PM2.5 exposure-sensitive, non-smoking COPD patients were distinguished by a relatively high hsa circ 0005045 expression, along with one group in a study (mMRC 0–1 (or CAT 10), and 2 exacerbations (or 1 exacerbation leading to hospital admission) in the past year), using machine learning models in a cohort of 327 COPD patients [77].

In a different study, CS reduced the levels of circRNA 0026344 and PTEN, which triggered autophagy and death in alveolar epithelial cells. Moreover, overexpression of circRNA 0026344 prevented CS extract (CSE)-induced autophagy and death in alveolar epithelial cells, but this obstruction was removed by upregulating miR-21 using a mimic. The authors’ findings showed that CS decreases the amounts of circRNA 0026344 in alveolar epithelial cells, which sponges miR-21 to suppress the miR-21 target, PTEN, which, in turn, activates ERK and encourages autophagy and death, causing emphysema. In order to control the PTEN/ERK axis in emphysema, circRNA 0026344 sponges miR-21, which is linked to CS-induced autophagy and death of alveolar epithelial cells. In conclusion, this work revealed a new mechanism for CS-induced emphysema and offered knowledge that will be helpful for identifying the condition and treating it [78].

Finally, mounting data suggests that air pollution exposure during pregnancy might result in congenital abnormalities in the fetus. Researchers analyzed differentially expressed circRNAs in rat embryos exposed to high levels (>200 g/m^3^) of fine PM using circRNA sequencing. In the embryos exposed to air pollution, 25 and 55 circRNAs were found to be downregulated and upregulated, respectively, in comparison to the control embryos whose mothers were raised in clean air. Adrenergic signalling was disrupted by in-utero air pollution exposure, according to analysis of circRNA-coexpressed genes using Gene Ontology (GO) and the Kyoto Encyclopedia of Genes and Genomes (KEGG). Also, researchers determined that three circRNAs—circ 015003, circ 030724, and circ 127215—play a crucial part in the network of circRNA-microRNA interactions. These findings suggested that circRNA dysregulation might be an important factor in the emergence of illnesses linked to air pollution [79].

The impact of circRNAs on adrenergic signalling may affect how quickly respiratory illnesses develop. Adrenergic signalling is actually a part of the sympathetic nervous system, and its natural ligands, norepinephrine (NE) and epinephrine, control a wide range of autonomic processes that include the innate and adaptive immunological response. The majority of peripheral tissues, including the lung and secondary lymphoid organs, are innervated by NE, the main neurotransmitter released by post-ganglionic sympathetic neurons. As a result, the adrenergic signalling pathways are in direct touch with both the central and peripheral immune compartments and may have an impact on inflammation when it comes to asthma attacks brought on by viruses [80].

Long, non-coding RNAs are one type of non-coding genomic material. Noncoding RNAs that are longer than 200 base pairs are referred to as long noncoding (lnc) RNAs [81]. Although most of them include a poly-A tail, lncRNA biogenesis is comparable to that of protein-coding RNAs and mRNA, but they cannot be translated into proteins [82]. Enhancer RNAs, intergenic transcripts, and snoRNA hosts are all types of lncRNAs [83]. They have been found in practically every type of cell and serve as the primary regulators of a wide range of cellular processes, including cell division, cell cycle progression, cellular architecture, transcriptional regulation, nuclear-cytoplasmic translocation, and posttranscriptional regulation. They also influence the epigenetic control of gene expression [84,85].

According to a study by He et al., antigen-presenting, immune-related MHC I, and adaptive immunological response were the main foci of the lncRNA MHC-unique R’s expression in the lung tissues of rats exposed to air pollution PM. After the prediction of transcription factors, it was discovered that GATA3 might be used in conjunction with the precise sequence of the lncRNA MHC-R promoter region. APCs with the most effective antigen-presenting activity are dendritic cells (DCs), which are essential. In order to engage in the pathogenic mechanism of COPD caused by PM air pollution, authors demonstrated that GATA3/lncRNA MHC-R may regulate the immunological activities of DCs. This finding paves the way for an early diagnosis and course of COPD treatment [86].

Finally, research by Lin et al. revealed that exposure to CS and PM2.5 increased expression of lncRNA LCPAT1 in lung cancer. The pro-tumorigenic effects of CS and PM2.5 on lung cancer cells were significantly reduced by LCPAT1 knockdown [87].

## 7. Conclusions

This paper suggests how air pollution and, in particular, chronic exposure to smoke is related to an increased risk for airway diseases, at the base of which there is overexpression, or not, of specific miRNA profiles.

Our analysis of data reported in the literature allows us to understand how miRNAs represent not only inflammation biomarkers related to air pollution but also possible predictive therapeutic biomarkers and targets for several airway diseases, such as lung cancer, asthma, and COPD. Therefore, further studies are needed in terms of genomic research for new developments in this topical field, in light of the increase in air pollution in many parts of the globe and of the prevalence of related diseases.

## Data Availability

Data are contained within the article.
